# The Photo- and Phonosensitivity Avoidance Behavior Scales: Evaluating Clinical Utility in Pediatric Primary Chronic Headache

**DOI:** 10.3390/children11111338

**Published:** 2024-10-31

**Authors:** Allison M. Smith, Megan N. Silvia, Hannah Rogan, Alyssa A. Lebel

**Affiliations:** 1 Division of Pain Medicine, Department of Anesthesiology, Perioperative and Pain Medicine, Boston Children’s Hospital, Boston, MA 02215, USA; hannah.rogan@childrens.harvard.edu (H.R.); alyssa.lebel@childrens.harvard.edu (A.A.L.); 2 Division of Psychology, Department of Psychiatry, Harvard Medical School, Boston, MA 02215, USA; 3 School of Occupational Therapy, Massachusetts College of Pharmacy & Health Sciences, Boston, MA 02215, USA; megan.silvia@mcphs.edu; 4 Department of Anesthesia, Harvard Medical School, Boston, MA 02215, USA

**Keywords:** primary chronic headache, chronic pain, phonosensitivity, avoidance

## Abstract

**Background/Objectives**: Pediatric primary chronic headache disorders are often associated with sensitivities to light (photosensitivity) and sound (phonosensitivity) that may trigger or worsen headache pain. These sensory sensitivities may result in changes to activity participation or environmental modifications to avoid visual and auditory stimuli. Over time, avoidance behaviors can inadvertently increase functional disability, suggesting the importance of their thorough consideration. The PhotoSensitivity and PhonoSensitivity Avoidance Behavior Scales (PHOTO-SABS and PHONO-SABS, respectively) were recently developed and preliminarily validated to assist clinicians in evaluating such behaviors. This study aimed to confirm each of their factor structures in a new sample and enhance their clinical utility. **Methods**: A sample of 176 youth (aged 8–17) with a primary chronic headache diagnosis completed the PHOTO-SABS and PHONO-SABS as part of their multidisciplinary evaluation in a pediatric headache clinic. **Results**: Consistent with the previous validation, confirmatory factor analyses supported a two-factor model for the PHOTO-SABS and a single-factor model for the PHONO-SABS. Tertile groupings (low, moderate, high) provided the most appropriate clinical reference points. The relative change criterion (RCCrit) was established at 6.4 points for both measures. **Conclusions**: These findings confirm that the PHOTO- and PHONO-SABS are psychometrically robust tools for clinicians to evaluate sensitivity-related avoidance behavior and to monitor response to interventions in youth with primary chronic headaches.

## 1. Introduction

A 2024 systematic review and meta-analysis [1] indicated that the overall prevalence of chronic headaches (headaches persisting more than three months) in children and adolescents is remarkably high, at 25.7%, across chronic migraine with/without aura, chronic tension-type headaches, New Daily Persistent Headache (NDPH), and other chronic headache subtypes. In addition to the discomfort itself, chronic headaches in youth are responsible for considerable comorbid functional disability, reflected in impairment across physical, social, emotional, and academic domains [2,3,4]. Given the extensive economic burden, comorbidities, and impact on quality of life associated with chronic headaches [5,6,7], the ability to understand and accurately assess factors contributing to headache-related disability represents an important area of research.

When examining pediatric chronic headache, it is critical to examine not only the severity and impact of head pain itself but also those of its associated features, as these too can contribute to severe functional impairment and poorer quality of life [8,9]. One subset of associated features worth noting, considering the central nervous system’s role in chronic headache and chronic pain [10,11] and the potential for impact on functioning, is heightened sensitivity to non-noxious sensory stimuli (e.g., visual, auditory, olfactory, somatosensory) [12]. Though migraine is most often associated with these hypersensitivities (e.g., photophobia, phonophobia, osmophobia), more recent explorations of headache subtypes in children and adolescents reveal that such sensory sensitivities are present across pediatric chronic headache presentations. For instance, when comparing youth diagnosed with chronic migraine with/without aura, chronic tension-type headache, and NDPH, Reidy et al. (2020) [8] identified no clinically meaningful differences in associated features, including photophobia and phonophobia, or in degree of functional disability. Similarly, Strong et al. (2021) [13] reported that the overwhelming majority (85%) of pediatric patients with NDPH experience photophobia and/or phonophobia, as well as reduced activity levels (88%).

Historically, to explain the relationship between the experience of chronic pain and functional disability, researchers have turned to the Fear-Avoidance Model (FAM) [14]. This model emphasizes that pain-related catastrophizing, fear, and avoidance behavior are critical contributors to the cycle of pain-related disability. When an individual perceives a pain-associated stimulus as a threat, they may come to fear the stimulus and pain; such fears can precipitate avoidance. Thus, one may become increasingly disabled as attempts to avoid pain and its related stimuli generalize. In fact, in their pediatric application of the FAM, Simons and Kaczynski (2012) [15] noted that avoidance behavior is a “more proximal link to functional outcomes”, as compared to pain-related fear or pain catastrophizing, likely due to its behavioral nature.

The FAM can be applied to the relationship between the associated headache features of light/sound sensitivities and headache-related disability, as avoidance of sensory input predicts functional disability in youth with headached [9]. However, despite this understanding (i.e., that headache-related sensory sensitivities are relevant across subtypes and that their avoidance contributes to disability), until recently, there were no validated measures of avoidance behavior specifically in youth with chronic headaches. Over a decade ago, Simons and colleagues (2011) [16] validated the Fear of Pain Questionnaire—Child Report (FOPQ-C), a standardized, commonly used tool for assessing pain-related fear and avoidance in youth with chronic pain. When analyzing their findings by pain subgroup [17], the authors noted that numerous FOPQ-C items are based upon fear/avoidance of movement, rendering them less relevant to patients with chronic headaches, as compared to potential items about cognitive demand or sensory input.

Further, in the literature, often, either fear and avoidance are examined in parallel (i.e., within the same measure) or fear is utilized as a proxy for avoidance. However, some researchers [18] highlight that fear and avoidance are related but not synonymous constructs. In fact, given that excessive avoidance behavior most directly contributes to pain-related disability and deleterious outcomes, avoidance behavior should thus be examined separately from fear.

To address the gap in the literature on avoidance behavior related to sensory sensitivities in pediatric chronic headaches, our group recently developed [19] and provided initial validation [20] of the PhotoSensitivity Avoidance Behavior Scale (PHOTO-SABS) and PhonoSensitivity Avoidance Behavior Scale (PHONO-SABS). The first phase was multi-step measure development, whereby an expert panel generated relevant items and then a pilot sample was employed to establish the measures’ feasibility and understandability for completion. This entailed measure administration followed by cognitive interviews [19]. In the second phase, the measures were improved and empirically validated in a large, new sample, exploring individual item performance, factor structure, internal consistency, and item content. Both measures demonstrated strong internal consistency, construct validity, and criterion-related validity [20]. At present, both measures have been integrated successfully into outpatient clinics and intensive pain rehabilitation settings. However, there has not yet been replication to confirm the measures’ factor structures, nor has there been specific consideration of the measures’ clinical utility.

Thus, the purpose of this final phase was two-fold: (1) to confirm the factor structures of the PHOTO- and PHONO-SABS, and (2) to increase their clinical utility by delineating clinical reference points and the reliable change criteria (RCCrit) for each measure. This would finalize psychometrically sound tools for health care clinicians to evaluate functioning and measure response to intervention in youth with primary chronic headaches.

## 2. Materials and Methods

### 2.1. Participants

Youth (*N =* 176) aged 8–17 who sought initial evaluation at a multidisciplinary chronic headache program between May 2022 and May 2023 at an urban, pediatric hospital in the U.S. participated in the study. Much of the sample described their race as White, their sex assigned at birth as “female”, and their gender identity as “cisgender female”. These demographics are generally representative of the population of youth typically presenting to this tertiary clinic. See Table 1 below for more detailed demographic information.

Physician-assigned chronic headache diagnoses were based upon the International Headache Society’s International Classification of Headache Disorders, third editions (ICHD-3 [21]). Only patients with primary chronic headaches were included; patients with acute headache presentations or headaches secondary to another general medical condition were not included. Most participants had been coping with headaches for several years, with more than half reporting daily/constant headache.

Participant diagnoses included the following: chronic Migraine with/without aura (1.3 in the ICHD-3), Intractable/Chronic Tension-Type Headache (2.3 in the ICHD-3 [22]), and/or New Daily Persistent Headache (4.10 in the ICHD-3). There was also a notable subgroup of patients meeting criteria for chronic migraine who also met criteria for Intractable/Chronic Tension-Type Headache (often referred to clinically as “mixed” type [23]). While the latter is not an explicit ICHD-3 headache diagnosis, in pediatrics, chronic migraine and chronic tension-type headaches often co-exist and “may represent a distinct headache type” [23], which may also influence how youth with this presentation experience associated symptoms and respond to intervention.

### 2.2. Measures

#### 2.2.1. Pain Severity

The Numeric Rating Scale (NRS) [24] was utilized to evaluate pain intensity. Participants were asked to designate their typical pain level on a standard 11-point scale, ranging from 0 (indicating “no pain”) to 10 (indicating “the most pain imaginable”).

#### 2.2.2. Pain-Related Fear & Avoidance Behavior

The PhotoSensitivity Avoidance Behavior Scale (PHOTO-SABS) [20] is an 11-item self-report tool that evaluates behavioral responses to headache-related photosensitivity (i.e., perceived sensitivity to light). Each item asks participants to respond on a four-point Likert-type scale, ranging from “Never” to “Always”, with higher ratings reflecting greater avoidance or modification of the environment to accommodate photosensitivity. The PHOTO-SABS demonstrated a Cronbach’s alpha of 0.92 in the initial validation study, which also revealed two correlated but distinct subscales: challenges to participation and modification of environment.

The PhonoSensitivity Avoidance Behavior Scale (PHONO-SABS) [20] is a 12-item self-report tool that evaluates behavioral responses to headache-related phonosensitivity (i.e., perceived sensitivity to sound). Each item asks participants to respond on a four-point Likert-type scale, ranging from “Never” to “Always”, with higher ratings reflecting greater avoidance or modification of the environment to accommodate phonosensitivity. The PHONO-SABS demonstrated a Cronbach’s alpha of 0.92 in the initial validation study, which revealed a single scale for the measure (i.e., no subscales).

The Fear of Pain Questionnaire—Child report (FOPQ-C) [16] is a self-report inventory that assesses pain-related fear and associated avoidance behaviors in youth. Participants respond on a five-point Likert-type scale, ranging from “Strongly Disagree” to “Strongly Agree”. A total score is derived by summing the ratings, with higher scores indicating greater fear and avoidance related to pain. There are also two validated subscales, fear of pain and avoidance of activities, both of which were examined in this study.

#### 2.2.3. Headache-Related Impairment

The Headache Impact Test (HIT-6) [25,26] assesses youth perception of the specific impact of headache on daily life over the course of four weeks. Its six items evaluate the adverse impact of headache on various aspects of functioning, as well as the severity of headache pain. Participants respond using a five-point Likert-type scale, ranging from “Never” to “Always”. A total score is derived from the sum of the ratings, with higher scores reflecting a greater impact of headache pain on daily life.

The SChool REfusal EvaluatioN (SCREEN) [27] is an 18-item self-report tool designed to assess school avoidance and refusal in children and adolescents. Responses are scored on a five-point Likert-type scale, ranging from “Doesn’t apply to me at all” to “Applies to me completely”. Ratings can be summed to calculate a total score that can then be categorized by clinical reference points. There are in four subscales as well: Anxious Anticipation, Difficult Transition, Interpersonal Discomfort, and School Avoidance. The SCREEN School Avoidance (SCREEN-SA) subscale score was used in this study. Higher scores reflect greater school avoidance.

Pediatric Migraine Disability Assessment (PedMIDAS) [28] is a self-report inventory consisting of six-items that evaluate the impact of headache across several domains (e.g., school functioning, activities within the home, activities outside of the home). Participants indicate the number of days in the past three months that their functioning was affected by headaches. For the purposes of this study, the two items pertaining to functioning at home and functioning in activities outside of the home were examined at the individual level. Higher scores reflect a greater impact of headache on functioning.

### 2.3. Procedure

Before their initial multidisciplinary evaluation in the chronic headache program, youth and their caregivers are requested to complete standardized surveys through a secure electronic platform as a standard component of routine clinical care. These surveys gather information on pain, developmental and medical history, and psychological functioning. These clinical data are kept in a centralized data repository, the Chronic Pain Data Repository, which stores patient data for those treated in all of the institution’s pain-related clinical settings [29]. The repository has been overseen by an ongoing standardized research protocol approved by the hospital’s institutional review board (IRB) since October 2018. Researchers can formally apply to access de-identified data, by providing a data safety and monitoring plan, obtaining approval of the departmental scientific review committee, and signing a data use agreement. Importantly, while patients and families are strongly encouraged to complete these standardized surveys prior to their visit, their completion is not mandatory. Patients received all medically indicated treatments regardless of whether they completed the surveys.

### 2.4. Statistical Analyses

Data were analyzed with SPSS version 29 and SPSS-AMOS version 26. Descriptive statistics were performed to assess underlying assumptions of normality for all variables. To confirm the factor structures of the two original measures, the PHOTO-SABS and the PHONO-SABS, we performed a series of Confirmatory Factor Analyses. For the PHOTO-SABS, we also examined whether one factor was more parsimonious than the original two-factor structure. As the PHONO-SABS was originally determined to have a single factor, additional models were unnecessary. Goodness of model fit for each measure was assessed using a chi-square significance test (χ^2^), as well as the following indices of fit: χ^2^/df (<3.0 good, <5.0 acceptable), comparative fit index (CFI; >0.90 acceptable, >0.95 excellent), and root mean square error of approximation (RMSEA; <0.08 acceptable, <0.05 excellent) using their agreed upon benchmarks [30,31].

Based upon methods previously utilized to determine clinical reference points for similar measures pertaining to pediatric pain [32,33,34], we examined tertile and quartile groupings of PHOTO-SABS and PHONO-SABS scores. We sought to identify the most parsimonious classification systems for the PHOTO-SABS and the PHONO-SABS that aligned with statistically significant differences in pain-related fear and avoidance, headache-related impact on life, and school/activity functioning. These groupings would reflect distinct and clinically meaningful reference points for both measures. We followed the same procedure for each measure, so the process is described here once. We first classified PHOTO-SABS scores into three levels (i.e., low, moderate, high), using tertile groups based on score distributions. We then conducted a series of one-tailed, one-way ANOVAs with Tukey post hoc analyses to confirm the validity of the tertile classification system. To confirm that tertile reference points were clinically meaningful, we sought significant group differences in selected measures of construct and criterion validity. We examined the Tukey post hoc analyses, applying a Bonferroni correction for multiple analyses (*p* < 0.007) to account for potential inflation of the Type I error rate. Finally, to ensure that tertiles were indeed the most parsimonious grouping option, we re-classified PHOTO-SABS scores into four levels (i.e., none/minimal, low, moderate, high), using quartile groupings based on score distributions, repeating the process. We followed the same steps to determine clinical reference points for PHONO-SABS scores.

Finally, we calculated the reliable change criterion (RCCrit) to aid in evaluating potential changes in photo- and phonosensitivity avoidance behavior over time (e.g., before and after intervention). Following the procedure standardized in previous studies of similar measures [33,35], we calculated RCCrit from the standard deviation (*SD*) and the measure’s reliability estimate (i.e., Cronbach’s alpha, *rel*), using the following formula: RCCrit = (*SD* × √2 × √1 − *rel*) × 1.96. The RCCrits established here for the PHOTO-SABS and PHONO-SABS can be used to determine whether changes in photo- and phonosensitivity-related avoidance behavior scores are due to true changes (i.e., treatment response) or due to measurement error.

## 3. Results

### 3.1. Confirmatory Factor Analysis: PHOTO-SABS

**Figure 1:**
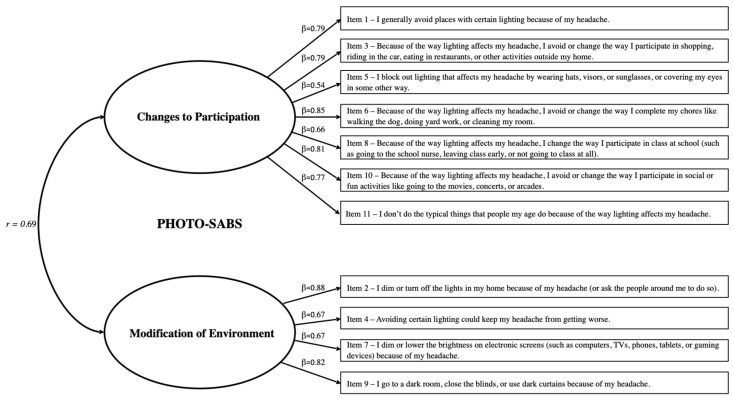
Confirmed factor structure and loadings of the PHOTO-SABS.

The PHOTO-SABS items were analyzed to confirm their contributions to the scale. None of the 11 items violated assumptions of normality (i.e., skew and kurtosis for all items were <2.0). Participant responses spanned the possible range (0–3) for all items. The CFA with items constrained to their original two-factor structure yielded a nearly acceptable model fit, though modification indices revealed covariance in the error terms that needed to be accounted for. Upon re-running the CFA with the same set of items, and with error covariances accounted for, the model demonstrated acceptable to excellent fit (χ^2^ = 84.13, *p* < 0.001, χ^2^/*df* = 2.16, CFI = 0.96, and RMSEA = 0.08). Figure 1 shows the final model with standardized regression coefficients/factor loadings (β) for the PHOTO-SABS subscales, which were strong, ranging from β = 0.54 to 0.88. By comparison, the single-factor model of the PHOTO-SABS showed a poor fit, confirming the necessity of including both factors. The PHOTO-SABS total score had a Cronbach’s α of 0.91, with a sample mean of 11.49 (SD = 7.72). The two subscales were each internally consistent (Changes to Participation α = 0.89, Modification of Environment α = 0.83) and intercorrelated, yet distinct (*r* = 0.69, *p* < 0.001). Total and subscale scores were normally distributed across the sample.

### 3.2. Confirmatory Factor Analysis: PHONO-SABS

**Figure 2:**
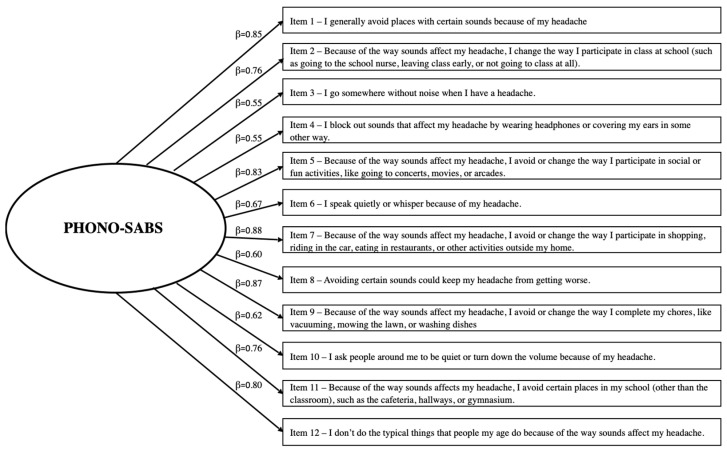
Confirmed factor structure and loadings of the PHONO-SABS.

In the same manner, the 12 PHONO-SABS items were analyzed to confirm their contributions to the scale. No items violated assumptions of normality (i.e., skew and kurtosis for all items were >2.0). Again, all participant responses spanned the possible range (0–3). The CFA, with items constrained to their original single-factor structure, initially yielded a nearly acceptable model fit, though once again, modification indices revealed covariance in the error terms that needed to be accounted for. Upon re-running the CFA with the same items, and with error covariances accounted for, the updated PHONO-SABS model demonstrated acceptable to excellent fit (χ^2^ = 116.60, *p* < 0.001, χ^2^/*df* = 2.33, CFI = 0.95, and RMSEA = 0.08). Figure 2 shows the final model with standardized regression coefficients/factor loadings (β) for the PHONO-SABS, which were strong, ranging from β = 0.55 to 0.88. The PHONO-SABS total score had a Cronbach’s α of 0.93, with a sample mean of 10.77 (SD = 8.78). Total scores were normally distributed across the sample.

### 3.3. Clinical Reference Points: PHOTO-SABS

PHOTO-SABS scores in the lowest tertile (i.e., scores between 0 and 7) represented low levels of photosensitivity-related avoidance behavior. Scores between 8 and 13 represented moderate photosensitivity-related avoidance behavior, and scores equal to or greater than 14 indicated high photosensitivity-related avoidance behavior. As illustrated in Table 2 displaying one-way ANOVA results, the three clinical reference groups (i.e., low, moderate, and high) significantly differed across all measures of construct and criterion validity (e.g., FOPQ-C Total, FOPQ-C Fear subscale, and FOPQ-C Avoidance subscale; HIT-6 Total; SCREEN-SA subscale, PedMIDAS Home and Activities items). Upon examining pair-wise comparisons, for the FOPQ-C total and subscale scores, all tertile groups were significantly different. For the HIT-6, the low group was significantly different from the moderate and high group (but the latter two did not differ). For the SCREEN-SA scale and the PedMIDAS-Home and PedMIDAS-Activities items, only the low and high groups were distinct from each other.

When repeating analyses using quartiles to define the groups, all ANOVAs remained significant; however, examination of pair-wise comparisons yielded far less sensitivity to between-group differences in the selected measures of construct and criterion validity. In particular, the none/minimal and mild groups did not significantly differ from one another across any of the measures examined. Thus, tertile-derived groups were chosen as the more appropriate and parsimonious clinical reference points.

Regarding demographic factors, there were no significant differences in participant age, sex assigned at birth, gender identity, or race across clinical reference groups, as measured by one-way ANOVAs and chi-square analyses. In terms of pain-related variables, there were no significant differences in photosensitivity-related avoidance behavior based upon headache diagnosis, time since headache onset, headache frequency, and duration of each headache episode. There were significant differences in headache pain intensity (*F* = 7.456, *df* = 2, *p* < 0.01). Inspection of pair-wise comparisons revealed that, while the high- and moderate-avoidance-behavior groups rated their pain intensity significantly higher than the low-avoidance-behavior group, the high- and moderate-avoidance-behavior groups were not significantly different from one another in terms of pain intensity.

### 3.4. Clinical Reference Points: PHONO-SABS

PHONO-SABS scores in the lowest tertile (i.e., scores between 0 and 5) represented low levels of photosensitivity-related avoidance behavior. Scores between 6 and 13 represented moderate photosensitivity-related avoidance behavior, and scores equal to or higher than 14 represented high photosensitivity-related avoidance behavior. As can be seen in Table 3 displaying one-way ANOVA results, the three clinical reference groups (i.e., low, moderate, and high) significantly differed across all measures of construct and criterion validity (e.g., FOPQ-C Total, FOPQ-C Fear subscale, and FOPQ-C Avoidance subscale; HIT-6 Total; SCREEN-SA subscale, PedMIDAS Home and Activities items). Upon examining pair-wise comparisons, for the FOPQ-C total and subscale scores, all tertile groups were significantly different. For the HIT-6, the low group was significantly different from the moderate and high group (but the latter two did not differ). For the SCREEN-SA scale and the PedMIDAS-Home and PedMIDAS-Activities items, only the low and high groups were distinct from one another.

When repeating analyses using quartiles to define the groups, all ANOVAs remained significant. However, as with the PHOTO-SABS, inspection of pair-wise comparisons in PHONO-SABS quartile groups yielded far less sensitivity to between-group differences in the selected measures of construct and criterion validity. Neither the none/minimal and mild groups nor the moderate and severe groups were significantly different from one another across the majority of the measures examined. Thus, tertile-derived groups were chosen as the more appropriate and parsimonious clinical reference points.

Regarding demographic factors, there were no significant differences in participant age, sex assigned at birth, gender identity, or race across clinical reference groups, as measured by one-way ANOVAs and chi-square analyses. In terms of pain-related variables, there were no significant differences in headache diagnosis, time since headache onset, headache frequency, and duration of each headache episode. There were significant differences in headache pain intensity (*F* = 6.818, *df* =2, *p* < 0.01). Inspection of pair-wise comparisons revealed that, while the high- and moderate-avoidance-behavior groups rated their pain intensity significantly higher than the low-avoidance-behavior group, the high- and moderate-avoidance-behavior groups did not significantly differ from one another in reported pain intensity.

### 3.5. Reliable Change Criterion (RCCrit)

Based upon the standard deviation (7.72) and the reliability estimate (0.91) of the PHOTO-SABS total score, the RCCrit is as follows: (7.72 × √2 × √1 − 0.91) × 1.96 = 6.4. Thus, changes in PHOTO-SABS total scores of more than 6.4 can be regarded as reliable changes (e.g., reductions if examining pre- to post-treatment intervention studies) in photosensitivity-related avoidance behavior. Similarly, based upon the standard deviation (8.78) and the reliability estimate (0.93) of the PHONO-SABS total score, the RCCrit is as follows: (8.78 × √2 × √1 − 0.93) × 1.96 = 6.4. Thus, changes in PHONO-SABS scores of more than 6.4 can be regarded as reliable changes (e.g., reductions if examining pre- to post-treatment intervention studies) in phonosensitivity-related avoidance behavior.

## 4. Discussion

This study represents the final phase in the validation of the PhotoSensitivity and PhonoSensitivity Avoidance Behavior Scales (PHOTO-SABS and PHONO-SABS). In the current phase, the authors sought to solidify the prior development [19] and validation [20] of these measures by confirming the scales’ psychometric properties, including internal consistency and factor structure. Furthermore, the authors also sought to expand the measures’ clinical utility by establishing clinical reference points and determining the reliable change criterion for each measure. These findings will allow clinicians to more clearly interpret the meaning of various scores on these measures as well as to assess clinically meaningful changes after intervention in youth who experience visual and auditory sensitivities with their primary chronic headaches.

### 4.1. Review of Findings

The 11-item PHOTO-SABS retained its same two-factor structure as the original measure validation study [20], with subscales measuring Changes to Participation and Modification of Environment. The total score and subscale scores of the PHOTO-SABS demonstrated strong internal consistency and strong factor loadings therein. Similarly, the 12-item PHONO-SABS retained its same structure, a single factor with no subscales. The total score of the PHONO-SABS exhibited excellent internal consistency and strong factor loadings.

The importance of having dedicated, specialized psychometrically sound assessment tools to guide research endeavors, particularly in pediatric headache, cannot be understated. These findings confirm, in a novel sample, what previous work has demonstrated: the PHOTO-SABS and PHONO-SABS are feasible, acceptable, sound, valid measures of previously understudied phenomena in pediatric chronic headaches. As noted previously, prior to the development of the PHOTO-SABS and PHONO-SABS, not only were there no validated measures of sensory sensitivity-related avoidance behavior, but there was also little investigation into photo- and phonosensitivity in pediatric headache and related behavior, beyond the presence/absence of the symptom. As researchers and clinicians learn more about the role of central sensitization [10] across pediatric chronic pain conditions, it appears crucial to understand the associated symptoms that may also contribute to functional disability. Further, while there are measures of pediatric pain-related fear and avoidance (e.g., FOPQ-C [16]), the PHOTO-SABS and PHONO-SABS explore beyond avoidance of movement and of pain to capture nuanced aspects of the pediatric chronic headache experience.

The key indices of clinical utility established here, clinical reference groups and an RCCrit for each measure, can further aid in the interpretation and application of PHOTO-SABS and PHONO-SABS scores. The clinical reference groups (tertiles labeled low, moderate, and high) were robustly sensitive in detecting differences across all measures of construct and criterion validity, with medium to very large effects across all variables of interest (η^2^ = 0.06–0.28 for the PHOTO-SABS and 0.06–0.27 for the PHONO-SABS). At the pair-wise level, this demonstrated the need for global measures of avoidance and functional impact but was less apparent for domain-specific items. Here, there were fewer significant differences between the low/moderate and moderate/high groups, suggesting that at the level of specific domains (e.g., school, home, social life/activities) in avoidance behavior scores may present more dichotomously. This is consistent with the smaller effects here vs. larger effects with measures more directly assessing avoidance behavior and global impact. Ultimately, these findings suggest that the clinical reference groups are applicable in research settings in cross-sectional research studies to classify patients as having low, moderate, or high levels of sensitivity-related avoidance behavior. They can also be used in clinical assessment to not only enhance understanding of the headache experience, but also to match patients with appropriate interventions and stratify patients into the suitable level of care. For instance, patients with high scores on the PHOTO- and PHONO-SABs may necessitate targeted desensitization interventions such as those offered in more intensive pain rehabilitation programs. On the other hand, those with low scores may benefit from education alone, perhaps on the Fear-Avoidance Model (FAM) of Chronic Pain [14]. Moreover, the RCCrit calculated for each of the two measures can be used to assess clinically meaningful change, an area often overlooked in the developmental of new measurement tools, particularly in pediatric chronic pain [33]. However, the potential applications of the RCCrit are innumerable, as it allows researchers and clinicians to determine whether the changes observed in treatment outcome studies and in day-to-day practice are clinically meaningful.

### 4.2. Implications for Clinical Practice

The PHOTO-SABS and PHONO-SABS are practical measures that can be administered in under three minutes, making them highly feasible for routine clinical use and in research settings [19]. Their ease of administration and interpretation allows for utilization by a wide range of health care professionals, across disciplines and with varying degrees of specificity in their practice. For instance, data gleaned from the PHOTO-SABS and PHONO-SABS have clinical relevance for care provided by headache specialists, such as pain physicians, neurologists, psychologists, and allied health practitioners (e.g., occupational and physical therapists). Clinicians are encouraged to incorporate the PHOTO-SABS and PHONO-SABS into their initial evaluations to assess avoidance related to sensory sensitivities, which may otherwise not be identified through closed-ended, dichotomous questions, such as, “Do you have sensitivity to lights or sounds?” At the same time, the PHOTO-SABS and PHONO-SAB can also be used by primary care physicians, who are often the first-line providers helping patients to assess persistent headaches. These measures can be implemented as brief screening tools to help pediatricians detect patients who may benefit from further neurological evaluation and/or specialized headache-related care. In fact, such tools may provide pediatricians with the data needed to justify a multidisciplinary approach to care for chronic headaches.

The PHOTO-SABS and the PHONO-SABS also complement the existing assessment tools typically included in headache clinic batteries, such as the HIT-6 [25,26], PedMIDAS [28], and FOPQ-C [16]. The addition of measures assessing the impact of sensory sensitivities on functioning facilitates a more comprehensive evaluation of the headache experience. By revealing nuanced and typically subtle avoidance behaviors, the PHOTO-SABS and PHONO-SABS provide valuable insights that can enhance treatment planning across disciplines and inform the selection of targeted interventions, ensuring a client-centered and individualized approach to care.

### 4.3. Limitations

This study is not without limitations, which warrant careful consideration. While the sample demographics in this study generally align with youth patients presenting to this tertiary multidisciplinary headache clinic in the Northeast, the sample was notably homogenous. Future research should aim to diversify sample characteristics, specifically focusing on sex assigned at birth, gender identity, race, and ethnicity, to enhance the generalizability of these findings and ensure that they reflect the experience of all youth with primary chronic headaches. Moreover, due to the self-reported nature of the PHOTO-SABS and PHONO-SABS, responses are inherently subject to potential response bias and the limited introspective capacity of respondents, particularly younger participants. To mitigate these risks, it is advisable to pair self-reported measures with clinical observations from clinicians and/or caregiver input. Future studies might also explore the development of separate caregiver proxy measures to complement youth self-reports. Additionally, it is important to acknowledge that data were collected electronically prior to the clinic visit, requiring respondents to engage in cognitive activity and interact with a screen. Particularly for individuals with photosensitivity, caregiver assistance to complete the measures may have introduced bias or influence upon youth responses. Further investigation of this measure could consider utilizing multiple methods of data collection (e.g., electronic, pencil/paper, or verbal) and/or data collection within the clinic setting to minimize this limitation and better accommodate diverse respondent needs.

### 4.4. Future Directions

The conceptualization of this measure development study emerged from an identified gap in the literature: no existing measures had been available to assess avoidance behavior in youth with primary chronic headaches who experience photo- and phonosensitivity. Following the development and initial validation of the PHOTO-SABS and PHONO-SABS, these tools have already been successfully integrated into multidisciplinary headache care and intensive interdisciplinary pain rehabilitation settings. With the measures’ clinical utility substantiated in this study, researchers now have valuable, methodologically sound resources to formally assess clinical changes in avoidance behaviors related to photo- and phonosensitivity. Continued application will help to disseminate their utility across settings and disciplines, particularly in the initial assessment and in setting treatment goals for functional rehabilitation. Now, with clinical reference points and the reliable change criterion, a novel application for these measures is the assessment of meaningful change in sensitivity-related avoidance behavior during and after participation in interventions focused on desensitization.

There are myriad potential extensions of the current measures, which could include exploring differences in sensitivity-related avoidance behavior across pediatric headache presentations. While photo- and phonosensitivity are typically most associated with pediatric migraine [36], anecdotally in the clinic and as evidenced in our three measure validation studies, it appears that such sensitivities are reported to be very much present in all primary chronic headaches experienced by youth. Understanding any nuances in those experiences could help to further individualize treatment approaches and identify potential barriers to functioning. Relatedly, another extension of these findings could be to explore sensory sensitivity-related avoidance behavior in non-headache pain conditions. Given the role of central sensitization of the nervous system in all manifestations of chronic pain [37], it is possible and even likely that youth with other types of persistent pain conditions could also experience sensory sensitivity-related avoidance behavior that contributes to their functional disability.

Replication of these validation studies and additional examination of their use in more diverse samples (with regard to demographics and headache characteristics) is a future direction to consider, as noted earlier. Further, given that primary chronic headaches can present and persist across the lifespan, future research could adapt and validate these measures in young adults and adults. Modifications to the current tools is warranted, such as replacing school-related items with those that pertain to job or work environments, to ensure broader applicability in these age groups. This would not only extend the utility of the measures but also enhance their relevance in diverse settings beyond the pediatric population.

### 4.5. Conclusions

The PHOTO- and PHONO-SABS are theoretically driven, psychometrically validated, clinically useful measures for assessing sensory sensitivity-related avoidance behavior in youth with primary chronic headaches. These measures offer unique insights by capturing how photo- and phonosensitivity, beyond pain, can contribute qualitatively to avoidance behavior, ultimately impacting developmentally appropriate functioning across multiple domains. By establishing clinical reference points, clinicians can better interpret scores on these measures, further quantify the degree of sensitivity-related avoidance behavior, and understand its deleterious impact on participation in daily activities. Furthermore, in determining the reliable change criteria, clinicians can monitor changes in avoidance behavior over time, perhaps to understand parallel changes in functioning or to assess responses to various interventions (e.g., auditory and visual desensitization). The PHOTO-SABS and PHOTO-SABS now allow clinicians to measure and track meaningful changes in sensitivity-related avoidance behavior in both clinical and research settings to improve outcomes for youth with primary chronic headaches.

## Figures and Tables

**Table 1:** Self-reported demographic and headache history variables.

	*N*	%	Mean	SD
Age			14.73	2.45
Sex Assigned at Birth				
Female	132	75		
Male	44	25		
Gender Identity				
Cisgender Female	127	72.2		
Cisgender Male	43	24.4		
Non-Binary	2	1.1		
Transgender	1	0.6		
Not Reported	3	1.7		
Race				
White	134	76.2		
Black or African American	3	1.7		
Asian American	2	1.1		
Multiracial	4	2.3		
Not Reported	12	18.7		
Ethnicity				
Non-Hispanic	145	89.2		
Hispanic	19	10.8		
Chronic Headache (HA) Diagnosis				
Chronic Migraine (CM)	69	39.2		
New Daily Persistent Headache (NDPH)	41	23.3		
Mixed Type (CM with CTTH)	39	22.2		
Chronic Tension-Type Headache (CTTH)	19	10.8		
Diagnosis unavailable	8	4.5		
Chronic Headache Presentation				
Time Since Onset (Months)			42.9	62.75
Typical Pain Intensity Rating (NRS)			7.34	1.82
Frequency = Daily/Constant	89	50.6		

**Table 2:** One-way ANOVAs between PHOTO-SABS clinical reference groups and validity measures.

	Photosensitivity Avoidance Behavior Reference Group				
	Low	Moderate	High	*F*	η^2^
FOPQ-C Total score	30.48 ^b,c^	41.84 ^a,c^	51.75 ^a,b^	32.80 *	0.28
FOPQ-C Fear subscale score	15.67 ^b,c^	21.62 ^a,c^	26.57 ^a,b^	21.80 *	0.20
FOPQ-C Avoidance subscale score	14.81 ^b,c^	20.22 ^a,c^	25.17 ^a,b^	26.38 *	0.23
HIT-6 Total score	60.86 ^b,c^	64.55 ^a^	67.22 ^a^	18.47 *	0.18
SCREEN School Avoidance	8.14 ^c^	10.25	11.73 ^a^	9.39 *	0.10
PedMIDAS—Home Item	11.66 ^c^	19.28	30.43 ^a^	9.81 *	0.10
PedMIDAS—Activities Item	13.74 ^c^	16.38	28.46 ^a^	5.87 *	0.06

* *p* < 0.007. Within each row, group means with superscripts differ significantly from the other group(s) at *p* < 0.007, after applying a Bonferroni correction (e.g., a (low) is significantly different from b (moderate) and c (high)*,* and so forth). For PHOTO-SABS, sample size for each group was n = 58 (low), n = 55 (moderate), and n = 63 (high).

**Table 3:** One-way ANOVAs between PHONO-SABS clinical reference groups and validity measures.

	Phonosensitivity Avoidance Behavior Reference Group				
	Low	Moderate	High	*F*	η^2^
FOPQ-C Total score	31.24 ^b,c^	40.85 ^a,c^	52.66 ^a,b,c^	32.20 *	0.27
FOPQ-C Fear subscale score	16.22 ^b,c^	21.22 ^a,c^	26.76 ^a,b^	19.29 *	0.18
FOPQ-C Avoidance subscale score	15.02 ^b,c^	19.63 ^a,c^	25.90 ^a,b^	29.09 *	0.25
HIT-6 Total score	61.17 ^b,c^	64.54 ^a^	67.10 ^a^	15.07 *	0.15
SCREEN School Avoidance	8.91 ^c^	9.63	11.71 ^a^	5.75 *	0.06
PedMIDAS—Home Item	12.28 ^c^	20.83	29.12 ^a^	7.34 *	0.08
PedMIDAS—Activities Item	14.49 ^c^	16.26	28.69 ^a^	5.51 *	0.06

* *p* < 0.007. Within each row, group means with superscripts differ significantly from the other group(s) at *p* < 0.007, after applying a Bonferroni correction (e.g., a (low) is significantly different from b (moderate) and c (high), and so forth). For PHONO-SABS, sample size for each group was n = 58 (low), n = 59 (moderate), and n = 59 (high).

## Data Availability

The data presented in this study are available on request from the corresponding author. The data are not publicly available due to the privacy policies in place at the institution at which the data were collected.
